# Mnemonic vs. Executive Contributions to the N400: A Connectionist Approach to False Memories

**DOI:** 10.1007/s42113-024-00210-y

**Published:** 2024-08-02

**Authors:** Leo Sokolovič, Markus J. Hofmann

**Affiliations:** 1 Department of General and Biological Psychology, University of Wuppertal, 42119 Wuppertal, Germany; 2 Department of Neurology and Clinical Neurophysiology, Helios University Hospital Wuppertal, 42883 Wuppertal, Germany; 3 Faculty of Health, Herdecke University, 58488 Witten, Germany

**Keywords:** Event-related potentials, Recognition memory, False memory effect, Decision-making, Interactive activation model

## Abstract

The associative-read-out model (AROM) is an interactive activation model (IAM) of semantic and episodic memory, which has already predicted behavioral and neural data during recognition memory. The core AROM is a deterministic model containing the original IAM and a semantic layer, where long-term associations between words are defined by word co-occurrence statistics. Episodic traces on the other hand are reflected by the resting level activations of semantic word units. However, the AROM lacks an explicit decision layer. Here, we extend the AROM with a layer encompassing linear leaky competing accumulators, where evidence accumulation is driven by the activation from the episodic-semantic layer. We fit the resulting AROM + with individual episodic memory traces and decision parameters and show that it can account for accuracies and response times in a recognition memory task. Further it mechanistically shows that false and veridical memories are driven by the combination of the semantic and episodic memory signals. When exploring decision-related vs. mnemonic explanations of the N400, we find that the amplitude of the N400 is driven by episodic-semantic activation at frontal, central and posterior locations. This amplitude modulation is strongest over frontal electrodes supporting the view that the frontal FN400 is also driven by memory signals. In addition, the amplitude of the FN400 was influenced by the response criteria. In sum, the AROM + provides a convenient tool for disentangling episodic, semantic, and executive processes in recognition memory.

**Supplementary Information:**

The online version contains supplementary material available at 10.1007/s42113-024-00210-y.

## Introduction

The performance in recognition memory tasks can be summarized in terms of hit rates, i.e., the proportion of correct recognitions, and false alarms, i.e., the proportion of erroneous ‘Yes’ responses to new items (Stanislaw & Todorov, 1999). Studies have shown that the false alarm rate increases with the strength of the semantic associations of the learned stimuli to the lure. This observation has been named the false memory effect (Deese, 1959; Gallo, 2010; Hutchison & Balota, 2005; Roediger III & McDermott, 1995). Past research has shown that the false alarm rates for critical lures, i.e., new words with the strongest semantic association to learned words, range from 20 to 81%. In contrast, false alarm rates for new, semantically unrelated words lie between 8 and 38%. (Beato et al., 2012; Benjamin, 2001; Cadavid & Beato, 2016; Chen et al., 2008; Hutchison & Balota, 2005; Johnson et al., 1997; Kim & Cabeza, 2007; Nessler et al., 2004; Roediger III & McDermott, 1995; Trelle et al., 2017; Wolk et al., 2006). Compared to clear patterns of accuracy and false alarms, findings regarding response times are more heterogenous. For example, Tun et al. (1998) conducted a systematic investigation of response times patterns in a false memory paradigm and reported that hits and false alarms are equally fast (see also Johnson et al., 1997). More recent studies, however, reported more consistent findings and found that response times increase with increasing relatedness of critical lures to targets (Dennis & Turney, 2018; Nessler et al., 2001, 2004; Stuellein et al., 2016; Ye et al., 2016). Concretely, response times are fastest for targets and unrelated new items and slowest for critical lures. In the present study, we examine whether these latter findings may be explained by an explicit simulation of the decision processes.

Different mechanisms leading to the false memory effect have been described. The fuzzy trace theory proposes that the false memory effect is generated by the dynamics of verbatim and gist memory (Brainerd & Reyna, 1998). Accordingly, critical lures are accepted when recognition judgment is based on the gist information, rather than the contextual cues and surface form of a test item (Montefinese et al., 2015). An updated version of this theory (Brainerd & Reyna, 2018) suggests that the false alarms to critical lures in the false memory effect are not fictitious memories. The false memory effect is proposed to reflect a complementarity illusion. According to this illusion, some critical lures are not falsely remembered as being old. On the contrary, participants are thought to experience new items as both old and similar to old, i.e., new. This coexistence of the two incompatible states is generated by target recollection and context recollection, two processes subserving semantic familiarity. Erroneous target recollection leads to perceiving a new item as similar to an old item. Additionally, context recollection generates the feeling of an item being old (Brainerd & Reyna, 2018). The combination of these two recollection signals then leads to an erroneous ‘Yes’ response.

Kimball et al. (2007) proposed a model of the false memory effect, which is based on the extended search of associative memory model (Gillund & Shiffrin, 1984) and emphasizes the role of semantic associations both at encoding and recall. Thereby, the model also accounts for a boost in memory performance in studied old words. For false memories, the interference from semantic associations has also been suggested to arise from spreading activation between associated words and activation monitoring failures (Benjamin, 2001; Collins & Loftus, 1975; Roediger et al., 2001) . That is, long term semantic associations between studied words and critical lures increase mnemonic evidence for the critical lure. This activation is then mistaken for a true episodic memory trace due to a failure in activation monitoring. The effects of spreading activation and monitoring failures can contribute to the observed false memory effects during encoding, at test or on both occasions.

Importantly, it has been shown that not only semantic variables but also word frequency, orthographic and perceptual similarities have an independent and crucial contribution to the emergence of false memories in recognition memory (Clark & Gronlund, 1996; Coane et al., 2016; Collins & Loftus, 1975; Conroy et al., 2005; Gatti et al., 2022; Glanzer et al., 1993; Humphreys et al., 2023; Hutchison & Balota, 2005). Osth and Zhang (2023) for example showed that global orthographic similarity, calculated based on relative letter position within two words, had a comparable effect on false recognition errors as did a measure of semantic similarity, defined as a cosine of the angle between item vector representations derived from a word2vec model of a text corpus. Shiffrin et al. (1995) also investigated the effects of length (total number of words) and strength (average word strength in the category) of semantic and orthographic categories on false recognition. Their results show that category length but not strength led to an increase in false alarms, which was greater for critical lures compared to other distractors. Importantly, this effect was present for both semantic and orthographic categories. This shows that in studying false memory one must account for orthographic and semantic factors.

**Fig. 1:**
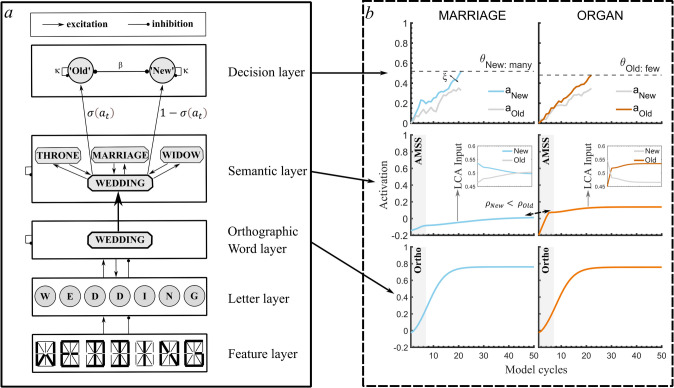
AROM+ model architecture. Panel **a** of the figure displays the model schematic for the stimulus ‘Wedding’. The right two figures in panel **b** show the time courses of the activations in the decision, semantic and orthographic layer for ‘Marriage’ (a new stimulus with many associates) and ‘Organ’ (an old stimulus with few associates). The bottom four layers in panel a correspond to previous IAMs (Grainger & Jacobs, 1996; Hofmann et al., 2011; McClelland & Rumelhart, 1981). The presented stimulus is first encoded as a pattern of activated and disactivated visual feature units. These then activate letter units, which in turn drive the corresponding orthographic units’ activation. The letter units, however, also suppress the activation of words which do not contain them. The word-identification signal from the orthographic layer is then propagated to the respective unit in the associative layer. There, the activation of the stimulus unit depends on the orthographic input, its resting level, and the spreading activation from the not presented but associated word units. In the decision layer, the Old response unit receives as input the sigmoid transformed momentary associative unit activation $\sigma {(a}_{t}$). The New response unit receives as input 1 - $\sigma {(a}_{t}$) and is thus driven by the absence of sufficient episodic-semantic evidence. In panel b, the time courses of model activations for two stimuli are shown. Grey areas in the orthographic and semantic layers mark the first seven processing cycles. From these the average orthographic (Ortho) and semantic activity (AMSS, Hofmann et al., 2011) were calculated for statistical analyses. New and Old words have practically identical orthographic activations, which shows that word identification proceeded equally fast for Old and New words. Therefore, the differences in the semantic activations are caused by the different resting levels (*ρ*) and the spreading activation from the associated stimuli. The inset ‘LCA Input’ plots display the time-evolving input for the decision layer units. They also demonstrate the effect of the sigmoid transformation. The activations of the response units in the decision layer and the decision criteria (*θ*) are shown in the top row. The variability of evidence accumulation due to decision noise (*ξ*) is also marked for the respond New unit. The evolution of the activations is governed by equations 1 and 2 (see the main text). They describe how the inputs, leak (*κ*), mutual inhibition (*β*) and decision noise (*ξ*) are combined to calculate the activity of accumulators in each processing cycle (${a}_{New}$, ${a}_{Old}$).

The false memory effect has also been modeled by an interactive activation and competition model (IAM, McClelland & Rumelhart, 1981; Rumelhart & McClelland, 1982), which is a computational model of context effects, originally designed for letter perception, but later extended to account for visual word recognition (Grainger & Jacobs, 1996; Hofmann & Jacobs, 2014; McClelland & Rumelhart, 1981; Perry et al., 2007; Rumelhart & McClelland, 1982). Hofmann et al. (2011) further extended the IAM to study the influence of semantic associations on recognition memory performance. In their model (see Fig. 1), each item’s orthographic activation serves as the word identification signal (Grainger & Jacobs, 1996), which is then used as input to the newly introduced associative-semantic layer (Hofmann et al., 2011). The dynamics of this layer represent the associative spreading activation among experimental stimuli (Hofmann et al., 2018). When modeling the influence of semantic associations on episodic memory performance one faces the non-trivial task of operationalizing and quantifying the strength of semantic association (Hofmann & Jacobs, 2014; Hofmann et al., 2011; Ratcliff & McKoon, 1994). Traditionally the strength of semantic associations was calculated based on association norms (Roediger III & McDermott, 1995). These give the frequency of a word being generated as a free association to a given cue word, e.g., how often “table” is produced given “chair” as cue. However, this approach has two important problems. One is a type of circularity in using the outcome of an association generating process to explain the inner workings of the association generating system (Hofmann & Jacobs, 2014). The other is the rather limited scope of association norms, which reflect only a few strongest associates and confound direct co-occurrence frequency and semantic association strength (Hutchison, 2003; Lucas, 2000; McNamara, 2005). To overcome the limitations of using associative norms and to quantify semantic association strength, Hofmann et al. (2011) used a text corpus approach. They built on the finding that semantic representations can be extracted from word co-occurrence statistics (Bullinaria & Levy, 2007). Thus, two words were taken to be semantically associated, if they occurred significantly more often together than alone in a sample of 43 million sentences (Quasthoff et al., 2006, see also the Stimuli section). In their experiment, they then examined whether the number of associates of each stimulus in the stimulus set affects recognition memory performance. They found that, compared to Old words with few associates (less than eight), Old words with many associates (at least eight) were more often correctly recognized. For New words, however, many associates compared to few associates led to an increased false alarm rate (see also Stuellein et al., 2016).

To study the contribution of semantic association strength to recognition memory, the memory signal used for recognition memory decisions was defined by Hofmann et al. (2011) as the average word unit activation in the semantic layer of the associative read-out model (AROM) in the first seven processing cycles. There were two motivations to define this average associative memory strength (AMSS) based on early model activations. The first one was to explain the frequently observed unequal variances of the memory signals of old and new stimuli (e.g., Green & Swets, 1974; Wixted, 2007). The second is based on the use of early (spreading) activation for the later decision mechanisms in the AROM’s predecessor, the multiple read-out model (MROM, Grainger & Jacobs, 1996).

Regarding the unequal variances, the AROM assumed the following: at modeling cycle 0, the resting levels of Old words are assumed to be higher than of New words in the episodic-semantic layer to represent that Old words have a higher memory signal. At cycle 1, since resting levels are positive, the first unit interactions and the spreading of activation in the semantic-episodic layer can take place. Therefore, all units receive excitation and inhibition from other units which are scaled by the last cycle’s activation (McClelland & Rumelhart, 1981, Eqs. 2 and 3). As this activation is higher for Old words, their “memory signal” variance is also necessarily higher than for New words (see Hofmann, 2011, p. 157f, for a simple demonstration with constant input; see also Fig. 8 in Hofmann & Jacobs, 2014, for a graphical illustration). This corresponds to the first assumption of the unequal variance model (Green & Swets, 1974). It also shows that the unequal variance assumption of the signal detection model follows directly from the AROM’s assumption of higher resting levels of Old words and the excitation and inhibition dynamics of the IAM layers. This is, however, necessarily true only in the first cycles. Depending on the amount of excitation and inhibition, the unequal variances may at some later cycle disappear. Therefore, early cycles often provide more realistic and stable predictions of the actual memory signals during recognition memory, much as MROM-based signal detection approaches to lexical decisions (Jacobs et al., 2003).

The second reason to base the AROM’s predictions on the initial seven cycles resulted from the MROM’s lexical decision mechanisms, which are based on early orthographic activation (Grainger & Jacobs, 1996). This early activation was proposed to be the first rapid estimate of lexical activation and has also been termed “fast-guess” mechanism (Braun et al., 2006). Its function is to modify two of the MROM decision criteria. On the one hand, high early lexical activity lowered the “yes” (lexical) decision criterion. On the other hand, when the early activation is high, it is unlikely that the stimulus is a nonword. Thus, the temporal deadline at which the “no” (lexical) decision is produced is postponed to later cycles (around cycle 20, cf. Grainger & Jacobs, 1996). This temporal deadline postponement also explained response times for “no” responses. This mechanism has also been discussed to account for prolonged response times to new items with many associates during recognition memory (Stuellein et al., 2016). In contrast to the MROM, we do not use a theoretical mechanism that couples early semantic activation with the temporal deadline. Rather, we fit the criteria based on the response time data.

In sum, the AROM was able to explain several recognition memory phenomena such as the greater memory signal strength variability for old items and the slope of the *z*ROC curves (Hofmann & Jacobs, 2014), while modeling both semantic and episodic factors in the false memory effect. The AROM represents a process model of recognition memory, which simulates the key perceptual, orthographic, semantic, and episodic processes required for recognition memory judgments. Furthermore, it also explains how the false memory effect results from these processes. In our present study, we further investigate the AROM’s capability to model recognition memory performance both in terms of behavioral data—accuracy and response times—as well as electrophysiological data.

The ability to model both cognitive and neural processes is a key benchmark for neurocognitive models (Forstmann & Wagenmakers, 2015; Hofmann & Jacobs, 2014). In this regard, the AROM has been able to predict event-related potentials (ERP). Using the same stimuli and experimental paradigm as Hofmann et al. (2011), Stuellein et al. (2016) found more negative amplitudes for New as opposed to Old words, and words with few as opposed to many associated items around 400 ms after stimulus presentation. This N400 effect for the number of semantic associates was most prominent at bilateral frontal, left central and posterior electrodes. As the N400 has been shown to be involved in lexical and semantic processing (Kutas & Federmeier, 2011; Lau et al., 2008) but also appears in studies of recognition memory (Rugg & Curran, 2007), Stuellein et al. (2016) suggested that the N400 is task dependent and might denote different cognitive processes in different tasks (for a similar argument see Bader et al., 2023). For example, its’ amplitude has been shown to reflect lexical factors such as the number and frequency of orthographic neighbors (Kutas & Federmeier, 2011; Laszlo & Federmeier, 2011). It is also modulated by lexical competition (Hofmann et al., 2008a, 2008b) and could therefore represent a special case of an N2 (Thompson-Schill & Botvinick, 2006; Yeung et al., 2004). A similar frontal ERP is observed in the frontal electrodes, which may represent the mnemonic signal in recognition memory tasks (Rugg & Curran, 2007).

The view that the functional interpretation of the N400 depends on the current task might offer a way forward in the debate on the interpretation of the frontal N400 (FN400) in recognition memory. In brief, a prominent stimulus-locked negative deflection at frontal electrodes with its peak at about 400 ms in recognition memory tasks has been proposed to represent a familiarity signal, tentatively generated in the medial temporal lobe (Bridger et al., 2012; Friedman & Johnson Jr., 2000; Kriukova et al., 2013; Rugg & Curran, 2007). However, this apparent difference in function between the FN400 and N400 is not consistently reported (Friedman & Johnson Jr., 2000). There is also a strong view that both ERP components reflect semantic processing and that there is no functional difference between them (Voss & Federmeier, 2011). However, by controlling for possible confounding of semantic priming and familiarity, Bridger et al. (2012) identified independent contributions of familiarity to the amplitude of the FN400. Further, Bader and Mecklinger (2017) argued that semantic priming and episodic familiarity additively influence the amplitude of the FN400. In a study on the FN400 during the study and test phases, Chen and colleagues (2014) concluded that the N400 and FN400 reflect semantic processing, both during study and at test. However, only the amplitude of the FN400 during the recognition memory test was correlated with higher old/new discriminability and faster response times. It has also been reported that the N400 for subsequently forgotten words tends to display more negative amplitudes during study thus implicating the N400 as a marker of episodic memory trace formation (Höltje et al., 2019; Meyer et al., 2007; Packard et al., 2020; Smith, 1993). Yang et al. (2019) also demonstrated that the amplitude of the FN400 reflects episodic evidence accumulation. On the other hand, the N400 in their experiment tracked both episodic and semantic familiarity in a decision independent manner, serving as an index of retrieval difficulty (cf. Brouwer & Hoeks, 2013; Brouwer et al., 2017). Bader et al. (2023) further suggested that the amplitude of the FN400 during recognition tracks the relative strength of episodic versus semantic familiarity.

There are challenges in dissociating the contributions of semantic and episodic memory to memory judgments. Firstly, episodic memory considerably overlaps with and can be viewed as a subcomponent of semantic memory (Tulving, 1985, 2002). Secondly, participants most likely engage both semantic and episodic memory systems when making a memory judgment (McClelland, 2013; McClelland et al., 1995). For example, Packard et al., (2017, 2020), showed that the use of semantic memory can improve episodic encoding of verbal material, especially if there is little opportunity to rehearse the material (cf. Howard & Kahana, 2002). The overreliance on semantic memory in episodic memory tasks can however increase the false alarm rate in recognition memory tests, i.e. induce the false memory effect (Deese, 1959; Nessler et al., 2004; Roediger III & McDermott, 1995). The explicit manipulation of semantic association strength in the false memory paradigm experiments and its’ dependence on word co-occurrence (Hutchison & Balota, 2005) enables one to test the proposed independent effects of semantic and episodic memory processes on the differences between the FN400 and the N400 (Bader & Mecklinger, 2017; Cadavid & Beato, 2016). For example, Nessler et al. (2004) used categorical word lists to compare participants’ performance on a classical list learning task and a false memory task. They found that the presence of the FN400 and an absence of the N400 was common to both tasks. Similarly, Greve and colleagues (2007) required participants to learn categorically associated word pairs in an associative memory task. They found an N400 component tracking the difficulty of semantic processing for semantically associated and unassociated word pairs. Like in Nessler et al. (2004), however, only the FN400 was sensitive to familiarity and thus indicated episodic memory content. Packard et al. (2017) provided further support for the role of the FN400 in memory by finding an FN400 at encoding of subsequently correctly recognized words. In a similar vein, encoding strategies have been shown to modulate the FN400 amplitude (Nessler et al., 2001, but see Beato et al., 2012).

However, not only the strength and kind of memory signal influence the FN400. It has also been shown to be sensitive to the decision criterion, i.e., the amount of mnemonic evidence needed for a response to be made (Stanislaw & Todorov, 1999). Specifically, the FN400 is more positive when participants raise their response threshold (Mecklinger & Bader, 2020; Rugg & Curran, 2007), suggesting that it reflects both mnemonic and executive processes (Andrew Leynes, 2021; Debruille, 2007; Kuchinke et al., 2011; Pires et al., 2014). These have also been suggested to play an important role in the false memory effect (Benjamin, 2001; Roediger et al., 2001) and depend on processing in the fronto-parietal cortices (Gallo, 2010; Kurkela & Dennis, 2016; Windmann, 2002; Ye et al., 2016). For example, Azimian-Faridani and Wilding (2006) explicitly manipulated criterion setting in a false-memory paradigm and reported more positive FN400 amplitudes in the conservative than in the liberal criterion condition (see also Andrew Leynes et al., 2019; Hill & Windmann, 2014).

This brief summary of the (F)N400 literature demonstrates the difficulties in mapping cognitive processes to composite measures of brain activity such as EEG data (Irish & Vatansever, 2020; Renoult et al., 2019). It also shows that the task-dependent modulation of the (F)N400 may depend on a combination of perceptual, orthographic, episodic, semantic, and executive processes. However, mechanistic neurocognitive models require the formalization of the relationships between cognitive processes, behavioral data and neurophysiology in a single model (Forstmann & Wagenmakers, 2015). They can thereby potentially disentangle confounded cognitive processes and their contributions to the N2/(F)N400, thus contributing to functional interpretations of neurophysiological data.

### AROM +: Architecture and Dynamics

To this end, we introduce an extended version of the AROM (Hofmann et al., 2011), which has been shown to successfully model key regularities of recognition memory (Glanzer et al., 1993; Hilford et al., 2015). However, it does so without a dynamic simulation of participants’ decision-making processes. We thus added a decision layer to the AROM to model both accuracy and response times in recognition memory. The updated AROM thus follows existing computational models of word learning paradigms, which simultaneously account for both memory and decision processes (Cox & Shiffrin, 2017; Osth & Dennis, 2015; Osth et al., 2018; Polyn et al., 2009). In decision-making research sequential sampling models have become the dominant models (Bogacz et al., 2006; Brown & Heathcote, 2008; Ratcliff & Smith, 2004; Tillman et al., 2020; Usher & McClelland, 2001; van Ravenzwaaij et al., 2012). These models describe the decision-making process as evidence accumulation over time, which results in a choice, when the accumulated evidence for one of the possible alternatives reaches a response threshold (Bogacz et al., 2006; Ratcliff & Smith, 2004). In choosing such a model for the AROM’s decision layer, we built on the work by Dufau et al. (2012), who modeled visual word recognition in the context of the multiple read-out IAM (Grainger & Jacobs, 1996). They fitted the leaky-competing accumulator (LCA) model of decision-making to data from a lexical decision task (Usher & McClelland, 2001). The LCA model is a sequential sampling model, which is conceptually related to the AROM as its competitive dynamics are governed by external inputs, activation decay and mutual inhibition between the decision units. Another consideration leading to the choice of LCA was the need for real-time processing of inputs, which is a core feature of IAMs. The model chosen for the decision layer therefore had to be able to cope with time-varying inputs. This ability has been established for the LCA (Tsetsos et al., 2011, 2012) and thus we chose it as the decision mechanism for the new AROM + .

The current implementation of the AROM + does not change the architecture and parameters of the original AROM (Hofmann et al., 2011). The only exceptions are the initial resting-level activations for the words in the semantic layer which were estimated for each participant (for an overview of the parameters see Supplementary Tables 1 to 3). As depicted in panel a of Fig. 1, the feature layer encodes the presented stimulus as a pattern of active and inactive visual feature units. These units then excite the letter units of those letters, which contain the visual features and inhibit those that do not. Likewise, the letter units excite the orthographic/word units which contain the letter and inhibit those that do not. At the same time, the activated word unit excites the letter units of its constituent letters. The activated orthographic unit also inhibits the other orthographic units. The activation of a word unit in the orthographic layer provides the word-identification signal, which drives the activation of the corresponding semantic layer unit. Importantly, resting levels of units in the orthographic layer reflect word frequency, thereby providing a measure of orthographic effects in word recognition. In the semantic layer, the activity of the currently presented word’s unit is determined by the word identification signal, the semantic resting level, and the spread of activation from significantly associated words. The amount of spreading activation is proportional to the semantic association strength, which reflects the strength of co-occurrence of pairs of words in a text corpus. As in the orthographic layer, activated units also inhibit other units in the semantic layer.

The novel decision layer comprises two response units which receive their input from the stimulus’ node in the semantic layer. The accumulation of mnemonic evidence in the AROM + is thus driven purely by the build-up of episodic-semantic activation, which in turn depends on the preceding layers and episodic resting level activation in the semantic layer. As noted above, the AROM + thereby accounts for crucial perceptual, orthographic, and episodic-semantic factors which influence recognition memory performance.

The LCA response units are assumed to receive positive inputs which sum up to one (Turner et al., 2016; Usher & McClelland, 2001). Positive inputs were achieved by using a sigmoid transformation of the activation of the stimulus’ unit in the semantic layer as input for the LCA units. That is, the input for Old response unit (${I}_{Old}$) was ${I}_{Old}=\frac{1}{1+{e}^{-{a}_{t}}}$, where ${a}_{t}$ denotes the activity of the stimulus’ unit in the semantic layer at cycle *t*. This also means that the inputs vary with time according to the activations in the associative AROM layer (see also Cox & Shiffrin, 2017; Polyn et al., 2009; Shinn et al., 2020). The sum to one constraint was then satisfied by setting the input for the New response unit to ${I}_{New}=1-{I}_{Old}$ (cf. Dufau et al., 2012). This transformation of the semantic layer activation is mathematically equivalent to the output of the softmax function for binary classification. Thus, the inputs to the decision layer represent the probabilities for Old and New response at each processing cycle. The mnemonic and decision-making processes in the AROM + are governed by the following free parameters:

- *κ*: leak or decay: the proportion of the response unit’s activation lost in a time step,
- *β*: mutual inhibition: the strength of the mutual inhibition between the decision units,
- *ξ*: decision noise: a random value added to each response unit’s activation at each time step,
- NDT: non-decision time: the part of the response time not used for the decision-making process (such as motor response preparation),
- resting levels for New (${\rho }_{New}$) and Old (${\rho }_{Old}$) items in the semantic layer, which reflect the greater episodic memory traces for Old than New items in the test phase of the recognition memory task and
- four criteria (*θ*) one for each of the four conditions obtained by crossing the factors Oldness (Old/New) and Number of associates (many/few). These criteria set the activation threshold at which the model produces a decision.

The changes in the decision layer units activations ($d{a}_{Old}, {da}_{New}$) in each processing cycle *t* are calculated according to Eqs. 1 and 2 (Usher & McClelland, 2001). For example, the change in the activity of the Old response unit at time step *t* is determined by two terms. The first one is the difference between the momentary input (${I}_{{Old}_{t}}$), the decay proportional to the activity in the previous processing cycle (${\kappa *a}_{{Old}_{t-1}}$) and the inhibition from the respond New unit (${\beta *a}_{{New}_{t-1}}$). This difference is multiplied with the scaling constant $\frac{dt}{\tau }$, which relates model time to real time. For the AROM + this constant is set to 0.06 so that each processing cycle represents 60 ms, as in the original AROM. The second term in the sum is the random noise scaled by the square root of the scaling constant.

1 $$d{a}_{{Old}_{t}}=\left({I}_{{Old}_{t}}-\kappa {a}_{{Old}_{t-1}}-\beta {a}_{{New}_{t-1}}\right)\frac{dt}{\tau }+\xi \sqrt{\frac{dt}{\tau }}$$

2 $$d{a}_{{New}_{t}}=\left({I}_{{New}_{t}}-\kappa {a}_{{New}_{t-1}}-\beta {a}_{{Old}_{t-1}}\right)\frac{dt}{\tau }+\xi \sqrt{\frac{dt}{\tau }}$$

The patterns of accuracy and response times thus reflect both the mnemonic evidence (coming from AROM) and the noisiness of evidence accumulation (in the decision layer). These are analogue to the drift and diffusion components of the drift–diffusion model (Ratcliff, 1978). Simulated response times (RT) were calculated as: $RT=NDT+{c}_{decision}*.06$ with ${c}_{decision}$ being the processing cycle at which the activation of an LCA unit reached the decision threshold.

In the present form, the AROM + uses four decision thresholds to reflect the different strengths of spreading activation driven by the semantic association strengths and the episodic resting level activations in our experimental data. The motivation for it is threefold. Firstly, Miller et al. (2011) investigated strategic aspects of false recognition and have shown that participants adapt their criteria in response to critical items. That is, participants were able to detect the semantic association strength of each item and raised their criteria accordingly.

Secondly, different criteria for Old and New responses are motivated by the different nature of accumulating information. While Old words are driven by the actual presence of a memory trace which was generated during the study phase of the experiment, the New words are driven by the evidence for the absence of the memory trace. Therefore, it is also possible that participants do not require the same amount of evidence to identify a word as Old compared to identifying it as New. In his model of recognition memory, Ratcliff (1978) for example also allowed the distances between the starting point of the diffusion process and the Old and New response boundaries to be different, i.e., have different decision thresholds.

Thirdly, the choice of four criteria is motivated empirically as we found that fitting the model with either one or two criteria did not lead to a correct description of the response times. In the supplementary materials we report a model fit using a single criterion. This one criterion model can still capture the false memory effect in accuracy but fails to account for the response times pattern (see Supplementary Fig. 4). The model with two criteria also failed to account for the response times patterns. The four criteria in the AROM + are thus a heuristic used to approximate the effects of the learning phase (Old/New) and the effect of number of associates on the response criteria.

### Study Aims

With the expanded AROM, we first aimed to investigate the AROM + ’s ability to account for response times and accuracy in a recognition memory task investigating the false memory effect. Our second aim was to establish a connection between measures of model dynamics and the N400 amplitudes. Simultaneously capturing behavioral and electrophysiological data is an important and challenging test for the model. Especially because of the proposed dissociation between the summary nature of response-time measures and the suggested process specific, real-time processing reflected in event related potentials (Kutas & Federmeier, 2011). Finally, the expected relationships between model dynamics and the N400 should help us gain further insight into the processes involved in the (F)N400. Based on the above summary of the (F)N400 literature, we expected that the (F)N400 will reflect both memory and memory-based decision-making processes.

Methods.

### Computational Methods

#### Optimization Procedure and Simulation Protocol

To fit the model to the data of each participant we adapted the cost function from Usher and McClelland (2001, Appendix E). The adapted cost function was calculated for each condition according to:

3 $$C=200*{\left({H}_{emp}-{H}_{model}\right)}^{2}+200*{\left({FA}_{emp}-{FA}_{model}\right)}^{2}+\frac{{\left(\langle {RT}_{emp}\rangle -\langle {RT}_{model}\rangle \right)}^{2}}{{SD}_{emp}^{2}}+{\left({SD}_{emp}-{SD}_{model}\right)}^{2}$$

where C = cost, H = hit rate, FA = false alarm rate, SD = standard deviation of response times and the $\langle \bullet \rangle$ denoting condition average.

Using this cost measure, the model was fitted to data using the ‘*patternsearch’* MATLAB (2023a) algorithm, which is appropriate for noisy objective functions, such as the cost function of the LCA. We used the *GSSSPositiveBasis2N* poll method using the classical algorithm with options set to use the complete poll, the *rbfsurrogate* search function and the s*uccess* poll order. The tolerance of the cost function was set to 0.001. For the LCA parameters we set the following boundaries: *κ*, *β*, *θ* were limited to the interval [0, 1]; *ξ* was restricted to values between 0 and 0.2; NDT was allowed to take on values between 0 and the minimum observed response time for the participant across all experimental conditions. The resting levels (*ρ*) for New and Old words in the associative layer were allowed to take on any value between 0 and 1. The resting levels of New words were constrained to be lower than the resting levels of Old words (${\rho }_{New}<{\rho }_{Old}$).

### Data

We used behavioral and electroencephalographic data from Stuellein et al. (2016). They showed that a high number of semantic associates increases recognition memory accuracy for Old words and decreases it for New words. Moreover, they also found response time effects, with many associates decreasing response times for Old words, while increasing rejection times for New ones. They also investigated the effects of the number of semantic associates and memory status of words (Old vs. New) on ERPs. While a detailed description of the experiment details can be examined in Stuellein et al. (2016), we here shortly summarize the most important information.

#### Participants

The experiment included 32 right-handed German participants. Three subjects were excluded from the EEG analyses due to low data quality resulting in 29 participants for the analyses (20 female, M_age_ = 22,5 years. SD_age_ = 3,39, range = 18–29). Participants were recruited from the University of Wuppertal community and received course credit, if they were students.

#### Experimental Task

The recognition memory tasks consisted of 160 German nouns, which were taken from Hofmann et al. (2011). The word sample was split into four conditions, each of 40 nouns, in a 2 × 2 design including the factors Oldness (Old vs. New) and Number of associates (few vs. many). The task consisted of a study and test phase. In the study phase participants were required to learn 80 words, which were presented in a pseudo-random order. In the test phase participants were presented with the 80 words from the study phase and 80 new words. Their task was to determine if the word was learned in the study phase. The participants were instructed to respond as fast and accurately as possible.

#### Stimuli

The stimuli were selected from the word corpus of the “Leipzig Wortschatz” project (Quasthoff et al., 2006)), which consists of 800 million (word-)tokens and 43 million sentences, primarily extracted from online newspapers (1992–2006, Hofmann et al., 2011). The selection of words ensured comparability of nouns in each experimental condition with respect to word frequency, number of orthographic neighbors, word length, imageability, emotional valence and arousal (Hofmann et al., 2011). Words selected for many associates condition had at least eight significantly co-occurring neighbors in the stimulus set, and the items in the few associates condition had less than eight. Thus, the stimuli effectively reflected associative confusability of each item with the others (Evert, 2005). For Old and New words in the final stimulus set, the number of significantly co-occurring items was four in the few and 14 in the many condition. The strength of semantic association between words was calculated by dividing the frequency of a pair of words co-occurring together in a sentence by the expected frequency when co-occurrences were based on chance alone. The strength of the excitatory semantic association between two words is then equal to the log10-transformed ${\chi }^{2}$ value of the within-sentence word co-occurrences statistic (if log10-transformed ${\chi }^{2}$ > 6.63, otherwise it is set to 0). That is, two words were considered associated when they occurred significantly more often together in the sentences than predicted by their single-occurrence frequency (Dunning, 1993).

#### EEG Recording and Processing

The electroencephalogram (EEG) was recorded from 32 electrodes, i.e. two references (A3, A4), a ground (FPZ) and 29 active electrodes (FZ, CZ, PZ, FP1/2, F7/8, F3/4, FT9/10, FC5/6, FC1/2, T7/8, C3/4, CP5/6, CP1/2, P7/8, P3/4 and O1/2). After preprocessing EEG data (described in supplementary materials), we calculated the mean N400 amplitudes for each participant’s trial. We analyzed nine electrodes grouped into three EEG regions: frontal (F3, Fz, F4), central: (C3, Cz, C4) and posterior (P3, Pz, P4). The ERPs for these electrodes are shown in Supplementary Fig. 2. The N400 time window was defined as the period between 300–400 ms post-stimulus onset, relative to a 200 ms baseline prior to stimulus onset. The mean N400 amplitudes for each experimental condition are displayed in Supplementary Fig. 3. The choice of electrodes and the N400 time window were chosen according to recent recommendations for replicability in N400 research (Šoškić et al., 2022).

### Statistical Analyses

We first calculated the mean accuracy and mean correct response times for all four experimental conditions (Old: few associates, Old: many associates, New: few associates, New: many associates). To address the first study aim, we calculated Pearson correlations between observed and simulated accuracies and response times. A high correlation between these variables would validate the model’s ability to generate similar mean accuracies and mean correct response times as in the observed data. In the next step, we performed item-level analyses for accuracy and response times. That is, we analyzed the influence of Oldness and Number of associates on the speed and probability of a correct response to a given item. We fitted a generalized linear mixed effects model (LMM) with Oldness and Number of associates as fixed factors. We also included random intercepts for participants and items nested within participants. For correct response times, we fitted a generalized LMM with the same fixed and random effects. However, to account for the skew in response time data, we used an inverse gaussian link function (S. Lo & Andrews, 2015). These analyses were also performed on the data produced by the AROM + . If the analyses of observed and simulated data would show the same effects, this would mean that the AROM + also captured item-level performance.

After assessing the correspondence between simulated and observed data, we explored the optimized cognitive parameters of the AROM + (i.e., *κ*, *β*, *ρ*, *θ*, *ξ*). Since criterion values were allowed to vary across experimental conditions, we tested if Oldness and Number of associates influenced criterion setting. We thus fitted an LMM and regressed both experimental factors and their interaction on the criteria. We also included random intercepts for each subject. Next, we investigated which cognitive parameters were associated with measures of recognition memory performance. Since model parameters were not normally distributed, we therefore calculated Spearman rank correlations (*ρ*) between the cognitive model parameters, accuracy, and correct response times. These analyses were meant to investigate if any of the cognitive model parameters has a direct relationship with the behavioral performance measures.

Finally, to address the second study aim, we used an LMM to investigate which measures of model dynamics influenced item-level (F)N400 amplitudes. To perform these analyses, we derived a set of predictor variables from the AROM + . These were meant to represent the most relevant moderators of (F)N400 amplitudes, as identified in the introduction. That is, we derived measures characterizing the orthographic (Kutas & Federmeier, 2011; Laszlo & Federmeier, 2011), episodic-semantic (Rugg & Curran, 2007; Stuellein et al., 2016), and executive processes (Hill & Windmann, 2014) in the AROM + . To represent the combined episodic and semantic memory signal, we calculated the item specific associative memory signal strength (AMSS, Hofmann et al., 2011) for all items for each participant. The AMSS is defined as the average activation of the stimulus’ unit in the associative layer in the first seven processing cycles. To capture the orthographic effects, we calculated the mean orthographic unit activation in the first seven processing cycles. The values of the AMSS and mean orthographic activation were obtained for each participant using the parameter values from the best fitting model. Following a reviewer’s suggestion, we also calculated the average associative and orthographic activation from the last seven cycles when the model activity has stabilized. Lastly, the four decision criteria were taken as measures of executive functioning. The influence of these variables on the (F)N400 was investigated in the frontal, central and posterior EEG Regions. The LMM for item-level (F)N400 amplitudes thus included the EEG Region, the mean orthographic activations, the AMSS, the four decision criteria and their interactions as fixed effects. It also included random intercepts for participants and items nested within participants. Since the predictors derived from the AROM + have a clear cognitive interpretation, any significant effects of these predictors would directly establish neurocognitive plausibility of the model. It would also provide additional insight into the functional interpretation of the (F)N400.

All statistical analyses were performed in R version 4.3.2 (R Core Team, 2023) using the packages lme4 (v. 1.1–34), lmerTest (v. 3.1–3), afex(v. 1.3–0), robustlmm (v. 3.2–2), car (v. 3.1–2), emmeans (v. 1.1.8), DHARMa (0.4.6), marginaleffects (v. 0.16.0), plotly (v. 4.10.3), ggplot2 (v. 3.4.4), and performance (v. 0.10.5). If the homoscedasticity assumption of the LMMs was violated, we computed robust LMMs. The formulas used in the glmer, lmer and rlmer functions are reported with the results below. For all LMMs nominal variables in the analyses, we used sum contrasts using the contr.Sum() function. Sum contrasts coding (also called effect coding) represents factors in the columns of the design-matrix so that the contrast weights of all factor levels sum to zero. It also assures that the intercept of the regression model represents the average over all factor levels adjusted for the effect of other predictors. For factors with two levels, sum coding compares the dependent variable means of each factor level (for a tutorial on sum coding see Brehm & Alday, 2022; for an introduction to LMMs for EEG analyses see Payne et al., 2015 and their supplementary materials). The *mixed* function from the apex package was used to calculate F tests for predictors in the model. In obtaining significance tests for F-values, we used the Kenward-Roger procedure to adjust the degrees of freedom. In post-hoc tests, we used the Holm correction for multiple comparisons to adjust the *p*-values. In the (F)N400 analyses we do not report pure EEG Region effects, but focus on the orthographic, episodic-semantic, and decision-making factors influencing the ERP amplitudes.

## Results

### Descriptive Statistics

**Fig. 2:**
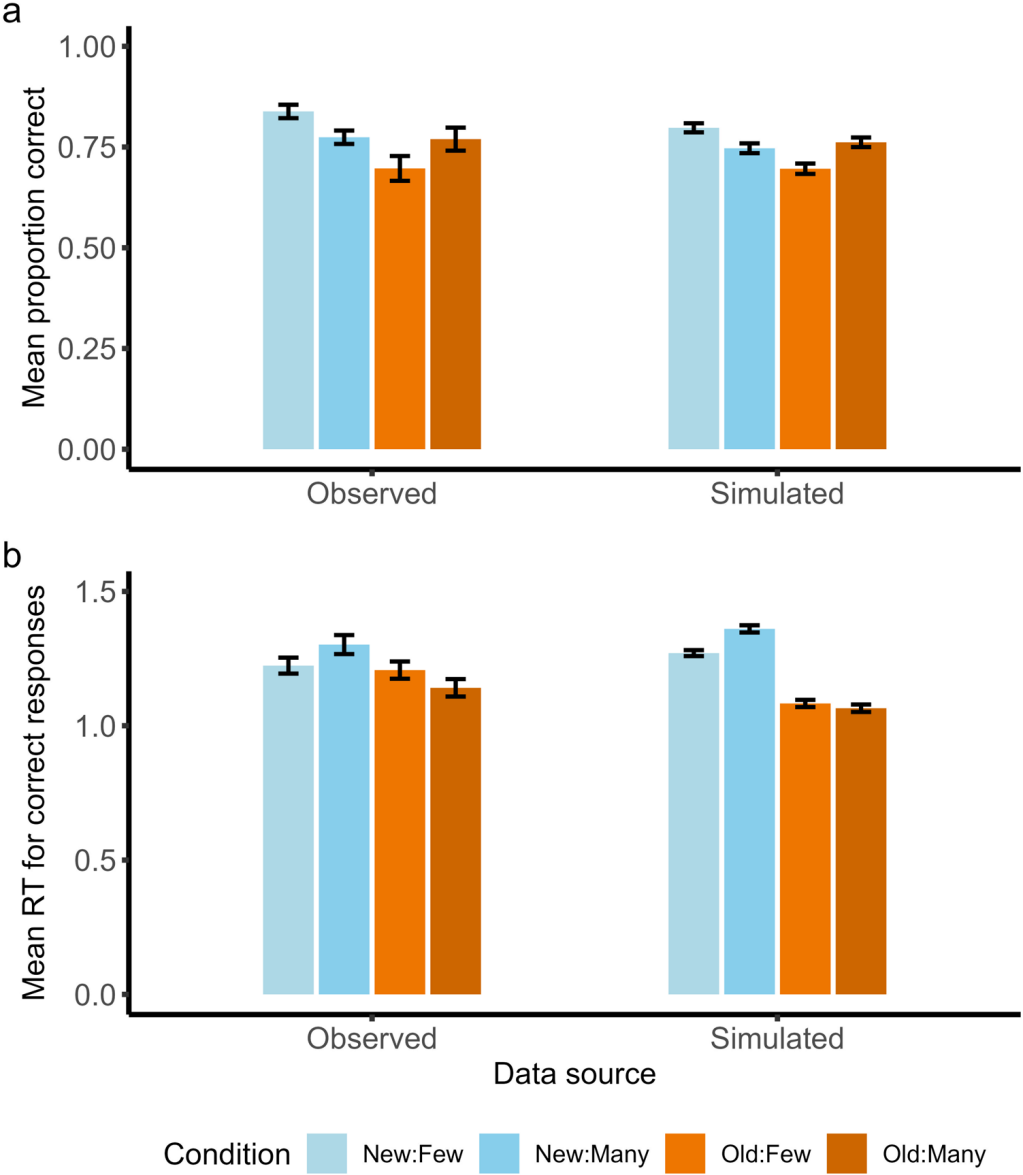
Performance (means and standard errors) in the four experimental conditions (Oldness: Number of associates) from behavioral and simulated data. **a**: average proportion of correct responses (hits and correct rejections). **b**: average response times in seconds for correct responses (hits and correct rejections)

Table 1 reports descriptive statistics for each experimental condition for behavioral and simulated data (see also Fig. 2). Regarding accuracy, Old words with few associates led to lower accuracies than Old words with many associates. For New words, accuracy was lower for words with many associates. The mean correct response times suggest that participants were faster to correctly recognize Old words with many associates than Old words with few associates. On the other hand, correct rejections of New words with many associates were slower than those of New words with few associates. The simulated data follow the same patterns of accuracy and response times. In general, the model reproduces the condition level accuracy data very faithfully (r = 0.91, R^2^ = 0.84, *p* =  < 0.0001) and performs well with respect to response times (r = 0.54, R^2^ = 0.29, *p* =  < 0.0001).

**Table 1:** Descriptive statistics of the behavioral data

		Behavioral data		Simulated data	
Oldness	Number of associates	RT in ms*	Accuracy*	RT in ms*	Accuracy*
Old	Many	1141 (32.5)	0.77 (0.029)	1065 (13.9)	0.76 (0.012)
	Few	1207 (32.1)	0.70 (0.031)	1083 (13.4)	0.70 (0.013)
New	Many	1302 (35.4)	0.77 (0.017)	1361 (13.4)	0.75 (0.012)
	Few	1224 (29.8)	0.84 (0.017)	1270 (11.1)	0.80 (0.011)

*RT: Response time for correct responses and Accuracy (proportion correct) both reported as mean (SE)

### Accuracy

We next performed item-level analyses of the influence of Oldness and Number of associates on probability of correctly recognizing or rejecting an item. We thus fitted a generalized LMM with a binomial link function. The model is summarized in Table 2 and shows that the probability of correct response depended on the interaction of Oldness and Number of associates. Post-hoc tests for the Number of associates show that Old words with many associates were more likely correctly recognized than Old words with few associates. For New words the pattern was reversed. Words with few associates were more likely rejected than words with many associates. These results show that having many associates facilitates the correct recognition of Old words but reduces the probability of correctly rejecting a New word. That is, for New words a high number of associates induces the false memory effect. The generalized linear mixed effects model for simulated correct responses is summarized in Table 3 and shows that the model reflects the response patterns of the behavioral data. It therefore successfully models the false memory effect.

**Table 2:** Generalized linear mixed model fit for correct responses

Fixed Effect	β	SE_β_	*z*-value	*p* – value
Intercept	1.387	0.101	**13.725**	< 0.0001
Oldness	0.261	0.063	**4.124**	< 0.0001
Associates	0.017	0.063	0.276	0.7826
Oldness: Associates	0.225	0.063	**3.575**	0.0003
Number of observations: 5120				
Random Effects				
	Variance	SD		
Stimulus (*n* = 160)	0.4232	0.6505		
Subject (*n* = 32)	0.1939	0.4403		
Post-hoc tests with Holm *p*-value adjustment				
Contrast	*M*_difference_	SEM	*z*-ratio	*p*-value
New: Few – Many	0.486	0.183	**2.658**	0.0079
Old: Few – Many	-0.416	0.174	**2.369**	0.0166

glmer formula: Correct ~ Oldness*Associates + (1|ID) + (1|Stimulus), family = binomial

Significant z-values are marked in bold

**Table 3:** Generalized linear mixed model fit for simulated correct responses

Fixed Effect	β	SE_β_	*z*-value	*p* – value
Intercept	1.146	0.073	**15.564**	< 0.0001
Oldness	0.119	0.034	**3.537**	0.0004
Associates	-0.011	0.034	-0.340	0.7337
Oldness: Associates	0.160	0.034	**4.761**	< 0.0001
Number of observations: 5120				
Random Effects				
	Variance	SD		
Stimulus (*n* = 160)	0.0068	0.0827		
Subject (*n* = 32)	0.1352	0.3677		
Post-hoc tests with Holm *p*-value adjustment				
Contrast	*M*_difference_	SEM	*z*-ratio	*p*-value
New: Few – Many	0.297	0.098	**3.046**	0.0023
Old: Few – Many	-0.343	0.092	**-3.709**	0.0002

glmer formula: Correct ~ Oldness*Associates + (1|ID) + (1|Stimulus), family = binomial

Significant z-values are marked in bold

### Response Times

We next turned to item-level correct response times. To analyze the influence of Oldness and Number of associates on empirical and simulated response times, we used generalized LMMs with an inverse gaussian link function. The model and the post-hoc tests for empirical response times are summarized in Table 4. The analysis shows that the empirical response times are influenced by the interaction of Oldness and Number of associates. The post-hoc tests showed no significant differences in response times between New words with few and many associates. However, Old words with few associates were more slowly recognized than Old words with many associates. The analysis of simulated response times (see Table 5 and Fig. 2) shows the same interaction. In conjunction with the LMM results regarding accuracy, the results of the response times LMM suggest that for Old words a higher number of associates leads to faster responses and higher probability of correct recognition. This modulation of response speed by the Number of associates is not present for New words. The results also show that the AROM + reproduces these effects.

**Table 4:** Generalized linear mixed model fit for correct response times

Fixed Effect	β	SE_β_	t-value	*p* – value
Intercept	1.290	0.041	**31.766**	< 0.0001
Oldness	0.049	0.014	**3.573**	0.0004
Associates	0.002	0.014	0.156	0.8764
Oldness: Associates	-0.032	0.014	**-2.292**	0.0219
Number of observations: 3941				
Random Effects				
	Variance	SD		
Stimulus (*n* = 160)	0.0097	0.0983		
Subject (*n* = 32)	0.0073	0.0855		
Residual	0.0806	0.2838		
Post-hoc tests with Holm *p*-value adjustment				
Contrast	*M*_difference_	SEM	*z*-ratio	*p*-value
New: Few – Many	-0.059	0.039	-1.521	0.1284
Old: Few – Many	0.068	0.039	**4.135**	0.0002

glmer formula: RT ~ Oldness*Associates + (1|ID) + (1|Stimulus), family = inverse.gaussian(“identity”)

Significant t-values are marked in bold

**Table 5:** Generalized linear mixed model fit for simulated correct response times

Fixed Effect	β	SE_β_	t-Value	*p* – value
Intercept	1.263	0.051	**24.781**	< 0.0001
Oldness	0.179	0.004	**41.727**	< 0.0001
Associates	-0.004	0.009	-0.480	0.6313
Oldness: Associates	-0.023	0.009	**-2.510**	0.0121
Number of observations: 3843				
Random Effects				
	Variance	SD		
Stimulus (*n* = 160)	0.0000	0.0000		
Subject (*n* = 32)	0.0117	0.1083		
Residual	0.0861	0.2935		
Post-hoc tests with Holm *p*-value adjustment				
Contrast	*M*_difference_	SEM	*z*-ratio	*p*-value
New: Few – Many	-0.054	0.033	-1.668	0.0954
Old: Few – Many	0.037	0.015	**2.433**	0.0150

glmer formula: RT ~ Oldness*Associates + (1|ID) + (1|Stimulus), family = inverse.gaussian(“identity”)

Significant t-values are marked in bold

### Model Parameters

The preceding analyses showed that the AROM + successfully reproduced the key aspects of behavioral performance. Table 6. shows the average optimized model parameters. Reflecting the constraints used in model fitting, resting levels of Old words were higher than resting level of New words. An LMM for decision criteria *θ* showed that only Oldness (t_(93)_ = 4.360, *p* =  < 0.0001) was a significant predictor, with criteria for New words being higher than for Old words (*M*_*difference*_ = 0.124, SE = 0.028).

**Table 6:** Means and standard errors of the model parameters

Oldness	Associates	$\rho$	$\kappa$	$\beta$	$\xi$	$\theta$	NDT
Old	Many	0.866 (0.037)	0.135 (0.029)	0.120 (0.028)	0.099 (0.006)	0.352 (0.039)	0.337 (0.037)
	Few					0.355 (0.032)	
New	Many	0.017 (0.007)				0.491 (0.025)	
	Few					0.465 (0.025)	

Note: *ρ*: resting level; κ: leak parameter; β: mutual inhibition; ξ: decision noise; θ: decision criterion; NDT: non-decision time in seconds

#### Model Parameters and Accuracy

We next explored if any of the cognitive model parameters had a direct relationship with accuracy. We therefore calculated the correlations between model parameters and accuracy scores at the interindividual level. The analyses showed a significant correlation between decision noise and overall accuracy (*ρ* = -0.224, *p* = 0.0109), suggesting that as decision noise increases, overall accuracy decreases. Decision criteria also correlated with accuracy for Old words with many (*ρ* = -0.558, *p* = 0.0018) and few (*ρ* = -0.554, *p* = 0.0018) associates, but not for New words with many (*ρ* = 0.057, *p* = 0.8430) or few (*ρ* = 0.147, *p* = 0.8430) associates. This suggests that executive processes involved in criterion setting only directly influence the accuracy for Old but not New words. Likewise, there were no significant correlations between *κ*, *β*, resting levels and accuracy.

**Fig. 3:**
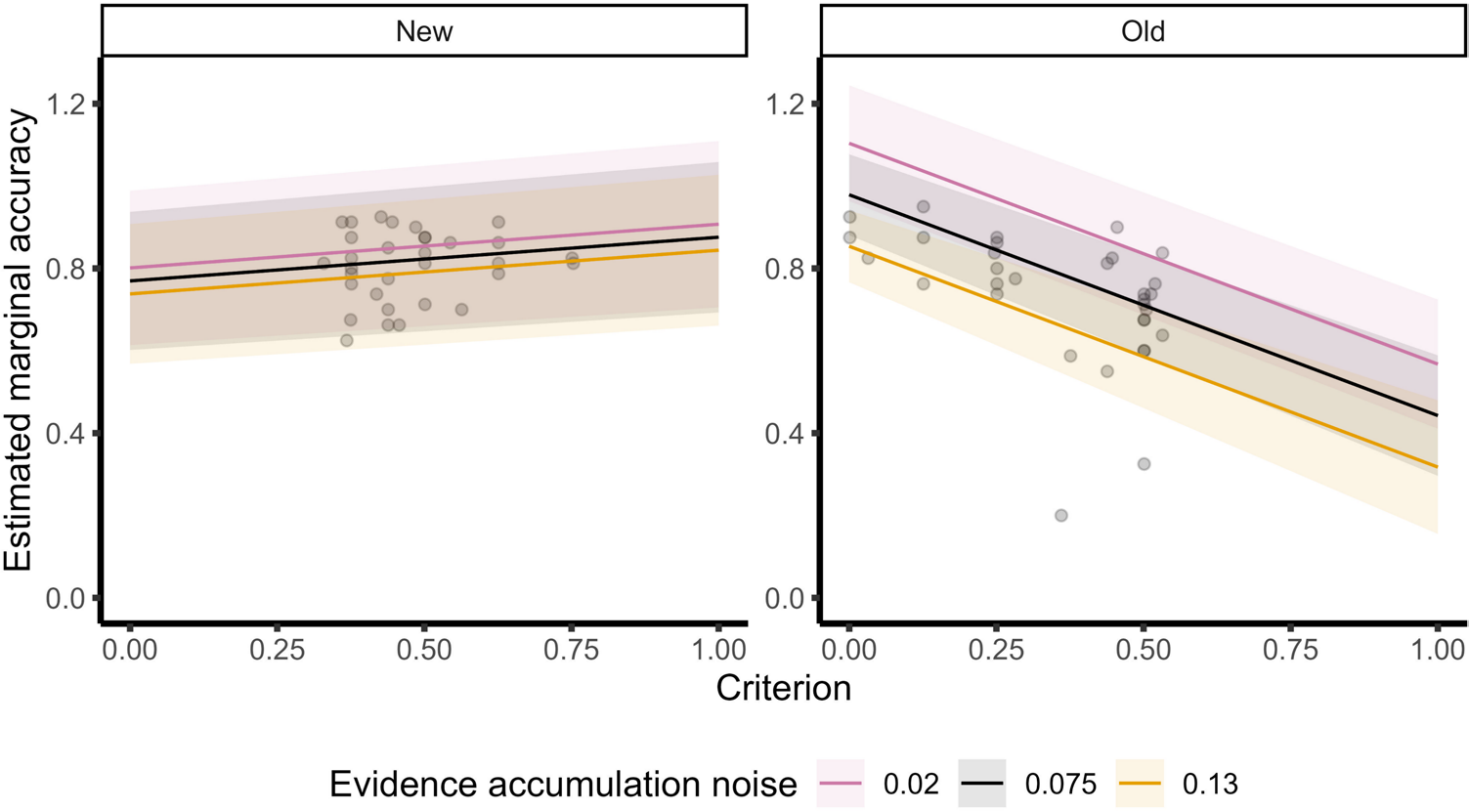
A plot showing the relationship between accuracy, decision noise and decision criteria for New and Old words. Regression lines show the relationship between decision criterion and estimated accuracy for three possible values of evidence accumulation noise. The chosen values correspond to the minimal and maximal evidence accumulation value in our sample and their average. The plot shows that participants with high decision noise and high criteria have lower estimated hit rates for Old words

To further investigate the relationship between accuracy, evidence accumulation noise and decision criteria, we first calculated each participant’s average criteria for Old and New words. We then fitted a LMM in which accuracy was regressed on the criterion and evidence accumulation noise within each level of Oldness. Finally, we plotted the estimated marginal means in Fig. 3. This plot shows that participants who had both high decision noise and high decision criteria had lower estimated accuracy. That is, increasing the decision threshold when the evidence accumulation was noisy impaired accuracy. As shown by the correlational analyses above this was the case for Old but not New words.

#### Model Parameters and Response Times 

We also investigated interindividual correlations between the cognitive model parameters and mean response times for correct responses. These analyses showed no significant correlations between response times and *ξ*, *κ*, *β* and resting levels. Decision criteria correlated with response times for Old words with many (*ρ* = 0.508, *p* = 0.0060) but not few (*ρ* = 0.326, *p* = 0.0683) associates. Likewise, there were no significant correlations between decision criteria and response times for New words with many (*ρ* = 0.177, *p* = 0.332) or few (*ρ* = 0.333, *p* = 0.1260) associates. These analyses show that the response speed for Old words with many associates is partly related to higher decision criteria.

In sum, the analyses addressing the first study aim show that the AROM + can reproduce behavioral findings. They further suggest that this ability cannot be simply explained by single parameters but that it stems from complex patterns of interactive activation and competition in the model’s processing layers.

### AROM + and the N400

**Fig. 4:**
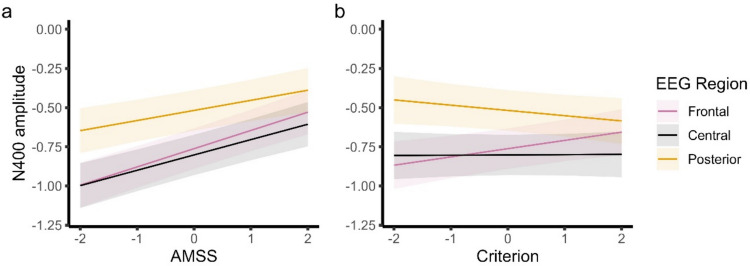
The EEG Region x AMSS (panel a) and EEG Region x Criterion (panel b) interactions. **a:** the N400 increases with increasing AMSS in all EEG Regions. Post-hoc analyses showed that this increase is smaller in the posterior EEG Region compared to frontal and central regions. **b:** the amplitude of the N400 also increases with increasing criterion, but only in the frontal EEG Region. The Criterion slopes in the central and posterior EEG Regions were not significantly different from zero

We now turn to AROM + ’s ability to predict the N400 amplitude. As described above, we derived measures of orthographic, semantic-episodic, and executive processes from the AROM + . We then used these measures as predictor variables in an LMM and regressed them on the N400 amplitudes. These were averaged for the each of the three electrodes of the frontal, central and posterior clusters for each item and each participant The analysis summary of main and interaction effects of this analysis is available in Table 7 (for the full regression results see Supplementary Tables 4 and 5). The analysis shows that the N400 amplitude was significantly predicted by the EEG Region, AMSS and the two-way interactions EEG Region x AMSS as well as EEG Region x Criterion (see Fig. 4). The results also showed that orthographic activation did not contribute to the explanation of (F)N400 effects in our recognition memory task. The same analysis but using the average orthographic and associative activation from the last seven processing cycles, i.e. the stable period in the AROM + , leads to identical results (see Supplementary Table 9).

**Table 7:** Main and interaction effects for the N400 linear mixed effects model

Effect	*df*	F-value	*p*-value
Intercept	(1, 28.784)	0.016	0.9012
EEG Region	(2, 7258.000)	**193.501**	< 0.0001
Orthographic activation (Ortho)	(1, 3602.296)	1.071	0.3008
AMSS	(1, 3628.621)	**29.265**	< 0.0001
Criterion	(1, 3408.537)	0.170	0.6803
EEG Region: Ortho	(2, 7258.000)	0.065	0.9370
EEG Region: AMSS	(2, 7258.000)	**5.332**	0.0048
Ortho: AMSS	(1, 3601.904)	0.645	0.4218
EEG Region: Criterion	(2, 7258.000)	**10.160**	< 0.0001
Ortho: Criterion	(1, 3601.886)	1.913	0.1667
AMSS: Criterion	(1, 3490.287)	0.902	0.3423
EEG Region: Ortho: AMSS	(2, 7258.000)	0.232	0.7930
EEG Region: Ortho: Criterion	(2, 7258.000)	1.054	0.3486
EEG Region: AMSS: Criterion	(2, 7258.000)	0.855	0.4253
Ortho: AMSS: Criterion	(1, 3601.797)	0.737	0.3898
EEG Region: Ortho: AMSS: Criterion	(2, 7258.000)	0.591	0.5536

rlmer formula: Amplitude ~ EEG Region * Ortho * AMSS * Criterion + (1|ID) + (1|ID:Stimulus); *p*-values were obtained using the Kenward-Roger approximation. Significant F-values are marked in bold

To investigate the significant effects, we performed post-hoc analyses. For the main effect of AMSS we calculated its estimated marginal slope, i.e., by how much the predicted N400 amplitude changes, when AMSS increases by one standard deviation. This change was calculated by partialling out the influence of all other model predictors. The analysis showed that the N400 amplitude significantly increases with increasing AMSS (*β*_*AMSS*_= 0.09, SE = 0.01, 95% CI = [0.07, 0.12], z = 6.48, *p* =  < 0.001).

The effect of AMSS was also involved in the EEG Region x AMSS interaction (Fig. 4a). To investigate this interaction, we first calculated the slope of AMSS in each EEG Region. This was done because of the reported differences between FN400 and the N400. Thus, it could be that the AMSS effect is significant in the frontal but not in central and parietal EEG Regions. These analyses, however, showed that the N400 amplitudes increased significantly with increasing AMSS in all regions (*β*_*frontal*_ = 0.12, SE = 0.02, 95% CI = [0.09, 0.15], z = 7.61, *p* =  < 0.001; *β*_*central*_ = 0.10, SE = 0.02, 95% CI = [0.07, 0.13], z = 6.40, *p* =  < 0.001; *β*_*posterior*_ = 0.06, SE = 0.02, 95% CI = [0.03, 0.09], z = 4.22, *p* =  < 0.001). We next tested if the strength of this effect changes from frontal to posterior EEG Region. To do so, we calculated consecutive differences between the AMSS slopes. That is, we tested if the differences *β*_*central*_-*β*_*frontal*_ and *β*_*posterior*_-*β*_*central*_ are different from zero. There was no difference between central and frontal slopes (*M*_*difference*_ = -0.018, SE = 0.012, z = -1.516, *p* = 0.1296). The difference between posterior and central region, however, was significantly different from zero (*M*_*difference*_ = -0.033, SE = 0.012, z = -2.738, *p* = 0.0124). Together, these analyses show that the N400 increases in all EEG regions with increasing semantic-episodic activation. However, the increase caused by the AMSS in frontal and central regions is significantly greater than in the posterior region. This pattern of N400 amplitudes suggests that the AMSS leads to an FN400 type signal in the frontal EEG Region.

We next turned to the EEG Region x Criterion interaction. We again estimated by how much the N400 amplitude changes when the Criterion increases by one standard deviation. These analyses show significant positive Criterion slopes in the frontal (*β*_*frontal*_ = 0.05, SE = 0.02, 95% CI = [0.02, 0.09], z = 2.82, *p* = 0.005) but not central and posterior regions (*β*_*central*_ = 0.00, SE = 0.02, 95% CI = [-0.03, 0.04], z = 0.09, *p* = 0.926; *β*_*posterior*_ = -0.03, SE = 0.02, 95% CI = [-0.07, 0.00], z = -1.80, *p* = 0.073). To investigate if the strength of this effect changes from frontal to posterior EEG region, we calculated consecutive differences between the Criterion slopes in each region. That is, we tested if the differences *β*_*central*_-*β*_*frontal*_ and *β*_*posterior*_-*β*_*central*_ are different from zero. We found significant differences between central and frontal (*M*_*difference*_ = -0.051, SE = 0.013, z = -3.958, *p* = 0.0002) as well as between posterior and central slopes (*M*_*difference*_ = -0.035, SE = 0.013, z = -2.741, *p* = 0.0061). The EEG Region x Criterion interaction thus shows that the N400 increases with increasing decision criteria and that this increase became steeper when moving from posterior to central electrodes. Finally, this increase was only statistically significant in the frontal EEG Region.

These analyses showed that the AROM + derived measures of semantic-episodic memory signal and criterion placement significantly influence the N400 amplitudes. The amplitude of the N400 increased with increasing AMSS in all EEG Regions, showing a global influence of the episodic-semantic memory signal. However, this effect was strongest in the frontal EEG Region. The results further suggest that processes also significantly contributed to the FN400 amplitude, since it increased with increasing decision criteria.

## Discussion

Our study had two major aims. First, we wanted to equip the AROM with a decision layer, which would allow it to mechanistically model the process of participants’ decision-making. To achieve this, we created the AROM + by integrating a new decision layer containing two response units. Their dynamic is described by the leaky competing accumulator model of decision-making (Usher & McClelland, 2001). This expansion enabled the model to capture behavioral data both in terms of accuracy and patterns of response times. At the same time the AROM + retained the ability of its predecessor to model complex interactions between experimental stimuli and their associates. To our knowledge, this is the first localist model of the false memory effect which mechanistically describes both spreading activation and decision competition in a single formal framework (but see Cox & Shiffrin, 2017; and Johns et al., 2012 for models using distributed item representations). The second aim of the study was to establish a connection between the measures of model activations and ERP data. In this regard, we demonstrated that the AMSS, a measure of the episodic-semantic memory strength, significantly contributes to the FN400 and N400 amplitudes in a recognition memory task. We also find support that the FN400 amplitude increases with increasing decision threshold.

The AROM + kept the ability of the original model to correctly predict higher accuracy for Old words and higher proportion of false alarms for New words. This effect results from the mechanism of spreading activation between word nodes (Collins & Loftus, 1975). Expanding on the insights provided by the AROM, the AROM + demonstrates that decision-making also plays a significant role. To successfully capture both accuracies and response times, we used four condition-specific decision criteria. These enabled the decision layer to flexibly respond to characteristic patterns and magnitudes of mutual interactive activation and inhibition of the stimuli from each of the experimental conditions (cf. Kroll et al., 2002; Scimeca et al., 2016). Indeed, a study using similar stimuli and experimental paradigm has shown that the four experimental conditions lead to different patterns of the BOLD response in frontal, midline, parietal and mesio-temporal regions, thus suggesting that underlying neural processes differ between the conditions (Kuchinke et al., 2013). Dennis et al. (2014) also suggested that fMRI activations of lateral and midline frontal brain regions signal the need for increased monitoring and conflict resolution demands in the false memory paradigm. In the AROM + this can be achieved by raising the response threshold, to allow for longer competition between response alternatives. Thus, we suggest that our condition-specific criteria are an amalgamate of monitoring and cognitive control of episodic-semantic retrieval (cf. Gallo et al., 2001; Hill & Windmann, 2014; Hofmann et al., 2008a, 2008b; Hofmann et al., 2008a, 2008b; Kuchinke et al., 2013; Yeung et al., 2004).

Interestingly, higher decision criteria correlated with worse accuracy for Old words but did not correlate with accuracy for New words, suggesting that cognitive control is particularly required for correctly recognizing Old words. Higher decision criteria within the AROM + framework allow longer competition between response units before making the final choice. Indeed, it has been suggested that conducting a prolonged retrieval process can be advantageous since it allows for a more robust recall process (Dennis et al., 2014). However, if the decision process is prolonged by increased decision criteria and if decision noise is high, it can sufficiently degrade the memory signal and thus lead to more errors. Indeed, our results show that noisier evidence accumulation correlated with lower overall accuracy. This finding conforms with the predictions of sequential sampling models (Ratcliff & Smith, 2004) and signal detection theory (Humphreys et al., 2023), as noisy evidence accumulation reduces discriminability. The idea that accuracy depends on the combination of decision criteria and noise in evidence accumulation is also supported by the multivariate surface plot in Fig. 3. It shows that participants with high decision thresholds and high decision noise had lowest accuracy for Old words. Importantly, such confounding of decision noise and criterion setting has also been shown to distort classical signal detection measures in perceptual decision-making (Mueller & Weidemann, 2008). The AROM + also explains the generation of false alarms. As described in the introduction, the semantic, excitatory connections between associated words lead to an increase in the corresponding unit’s semantic activation (cf. Kurkela & Dennis, 2016). This in turn, drives the activity of the Old response unit in the decision layer. False alarms can then result from a combination of the decision noise and the semantically mediated evidence accumulation in favor of the Old response (see also Ye et al., 2016). Further, the input to the Old response unit decreases according to the following episodic-semantic strength gradient: Old_many associates_ > Old_few associates_ > New_many associates_ > New_few associates_. Since among New words the input to the Old response unit is strongest for words with many associates, these words are more likely to be falsely recognized as Old.

The AROM + was also able to fit the patterns of response times observed in Stuellein et al. (2016). These analyses showed that whereas having many associates speeded up the correct recognition of Old words, the number of associates did not influence the speed of correct rejections of New words. However, there was a trend towards slower rejections of New words with many associates. This slowing cannot be completely explained by an increased response caution, since we found no difference between the decision criteria for New words with many and few associates. In the context of the AROM + , this slowing is most likely due to the different evidence accumulation rates for New words with many and few associates. These differences emerge because of the spreading activation dynamics in the semantic layer. That is, the activation of a New word’s unit in the semantic layer is determined by the number and strength of the semantic associations to Old words (see Supplementary Fig. 1). This leads to New words with many associates receiving more spreading activation. The resulting increase in semantic activation effectively decreases the input and evidence accumulation rate in favor of a correct rejection. Therefore, the AROM + offers a mechanistic explanation of the recent findings on response time patterns in the false memory paradigms, where increased similarity between old and new items leads to slower responses (Dennis & Turney, 2018; Nessler et al., 2001, 2004; Stuellein et al., 2016; Ye et al., 2016).

The second aim of the study was to establish a connection between the measures of model activations and ERP data. To that end, we calculated the average orthographic activation (Ortho) and the average associative memory signal strength based on the first seven model cycles (AMSS). In our statistical analyses of the N400 amplitude we included both Ortho and AMSS as well as the decision criteria and EEG Region (frontal, central, posterior) as predictors of the N400. The results showed that the episodic-semantic activation of the AROM + was a robust predictor of the N400 effect in all three regions. However, its influence is greatest over the frontal electrodes. This could indicate that the AMSS also interacts with executive processes. The effect of the AMSS in the frontal EEG Region could alternatively also reflect retrieval processes in the medial frontal lobes (Minxha et al., 2020) thereby supporting the interpretation of the FN400 as a marker of episodic mnemonic evidence accumulation in recognition memory (Greve et al., 2007; Nessler et al., 2004; Yang et al., 2019). This process may thus be contributing to (semantic) familiarity (Rugg & Curran, 2007; Stuellein et al., 2016). However, we also found that this FN400 is driven by higher response criteria. This is in line with findings from other studies on recognition memory (Azimian-Faridani & Wilding, 2006; Hill & Windmann, 2014) and the false memory effect (Andrew Leynes et al., 2019). The modulation of the FN400 amplitude by decision criteria suggests that executive processes contribute also to it. The presence of monitoring and cognitive control processes during recognition memory tasks are also supported by the fMRI study by Kuchinke et al. (2013) who reported the involvement of control-related frontal regions in a similar experimental paradigm (Kuchinke et al., 2013). Indeed, in the context of other tasks, it has already been suggested that the N400 is a marker of cognitive inhibition (Debruille, 2007; Pires et al., 2014) and that it reflects the processing in the left inferior frontal gyrus (Badre & Wagner, 2007; Stampacchia et al., 2018). Overall, our results show that the AROM + model disentangled the contributions of the spreading activation in semantic memory, the episodic-semantic memory strength and decision criteria to the amplitudes of the FN400 and N400 in recognition memory and the false memory effect (Benjamin, 2001; Cadavid & Beato, 2016; Gallo, 2010; Kurkela & Dennis, 2016; Roediger et al., 2001). On the other hand, none of our analyses offered any support for the role of orthographic processing in the generation of the N400 in the modeled recognition memory task. While orthographic variables have been shown to influence both the false memory effect (Gallo, 2010; Osth & Zhang, 2023) and the N400 amplitude (Kutas & Federmeier, 2011; Laszlo & Federmeier, 2011), we tentatively suggest that the lack of an orthography effect in our study may reflect the careful selection of experimental stimuli, which were balanced for word frequency and orthographic neighborhood (Grainger & Jacobs, 1996). To examine orthographic effects in the context of the AROM + , future studies could additionally manipulate word frequency and orthographic similarity of the stimuli (cf. Criss, 2010; Shiffrin et al., 1995). Alternatively, the obvious semantic confusability of the items may have engaged a (semantically-driven) deep evaluation strategy (Craik & Lockhart, 1972), thus lending support to the AROM’s assumption that study-test recognition memory performance is primarily driven by episodic-semantic activation. It is also possible that the way orthographic contributions to recognition memory were operationalized in the AROM + reduced their independent contribution to either phenomenon. Importantly, Supplementary Tables 6 to 8 show that the pattern of reported results does not change if the mean orthographic activation in the first seven cycles is not included as a predictor in the model.

The inclusion of decision-making dynamics in our model of recognition memory also has implications for the existing computational models of the N400, which emphasize its close connection to semantic processing (Brouwer & Hoeks, 2013; Brouwer et al., 2017; Laszlo & Armstrong, 2014; Laszlo & Plaut, 2012; Nour Eddine et al., 2022). While our model critically depends on the semantic associations and orthographic properties of the words through aggregation and integration of mnemonic evidence for a stimulus, it does so in the service of a decision layer, then produces a decision according to its own dynamics. The analyses of the model activations in conjunction with the N400 amplitudes enabled us to disentangle the influences of episodic-semantic, and decision-making processes on the amplitude of the (F)N400. In doing so, our results go beyond the discussion of familiarity vs. semantic processing when discussing the (F)N400. Our results suggest that this ERP component is not only task dependent (e.g., semantic familiarity vs. episodic memory signal) (Bader et al., 2023; Stuellein et al., 2016), but that it is also affected by different cognitive processes within a task, such as mnemonic strength and decision criteria. Further, we show that these processes can be disentangled using the AROM + . Finally, our results demonstrate that a computational model fitted to behavioral data can nevertheless lead to meaningful insights into ERP components (Kutas & Federmeier, 2011).

## Limitations

While the summary measures of model activations offered further insight into the functional interpretation of the N400, there are some limitations. One concerns the resting levels of New and Old words. While retrieved information in free recall and recognition memory contain both semantic and episodic memory traces (Berry et al., 2010; Osth & Dennis, 2015; Osth et al., 2018; Socher et al., 2009; Tulving, 1985), the same most likely holds for the encoding process. That is, when learning new information, both semantic and episodic traces are encoded into the new memory (Cox & Shiffrin, 2017; Kimball et al., 2007; Polyn et al., 2009). Therefore, it would be plausible to expect that the resting level of words varies according to both the item’s status (Old/New) as well as the number of its associates (many/few) (Healey et al., 2016; Hutchison & Balota, 2005). Thus, a possible extension of AROM would be an explicit model of the study phase to capture the influence of semantic associations at encoding. Another aspect of the decision-making layer which must be further explored is the dynamics of flexible criterion setting (cf. Dufau et al., 2012; Kuchinke et al., 2011). Trial-to-trial criterion changes could potentially allow the model to fit the data using fewer (e.g., one) but more flexible criterion. Furthermore, apart from trial-to-trial variability in criterion setting, there is also evidence for within trial criterion adaptation (Hill & Windmann, 2014). In computational models this can be implemented as collapsing decision boundaries, where the initial decision threshold is reduced in each processing cycle (cf. Cox & Shiffrin, 2017). These mechanisms need to be explored in a future version of the AROM. A practical computational disadvantage of the AROM + is the lack of a closed-form solution for the LCA, which makes model fitting very time consuming and stochastic (but see Lo & Ip, 2021). Alternative decision mechanisms, such as the generalized drift–diffusion model (Shinn et al., 2020) could be implemented instead of the LCA layer to make the model fitting more efficient. We also have the intuition that all IAMs could be expressed in a closed-form probability density function. This would enable efficient parameter estimation rather than setting them arbitrarily or using the original IAM parameters from McClelland and Rummelhart (1981).

## Conclusions

Here we show that the AROM + with an integrated decision layer can reproduce and explain recognition memory performance in terms of accuracy and response times. Moreover, the relationships between the summary measures of model activations and the ERP amplitudes suggest that the episodic-semantic activation drives the N400 at frontal, central and posterior sites and an additional FN400 in frontal electrodes. The FN400 is furthermore modulated by individually fitted response criteria demonstrating an executive contribution to this component.

## Supplementary Information

Below is the link to the electronic supplementary material.

Supplementary file1 (DOCX 808 KB)

## Data Availability

The simulation code, the behavioral and EEG data as well analyses scripts are available at: https://osf.io/fnyzg/.
