# Supplementary material for: Mnemonic vs. Executive Contributions to the N400: A Connectionist Approach to False Memories
Source: Comput Brain Behav. 2024 Aug 2;7(3):357–77. doi: 10.1007/s42113-024-00210-y (PMC13298628; doi:10.1007/s42113-024-00210-y)
Supplement: Supplementary file 1 — Supplementary file1 (DOCX 808 KB) [file 42113_2024_210_MOESM1_ESM.docx]

**Supplementary Materials**

# Model parameters

| Table 1. All excitation and inhibition model parameters of the AROM+ | | | | | |
| --- | --- | --- | --- | --- | --- |
| Excitation parameter α | | | | | |
| Layer | Feature | Letter | Orthographic | Associative | Response |
| Feature | 0 | .005 | 0 | 0 | 0 |
| Letter | 0 | 0 | .07 | 0 | 0 |
| Orthographic | 0 | .3 | 0 | .09 | 0 |
| Associative | 0 | 0 | 0 | .03 | 1 |
| Response | 0 | 0 | 0 | 0 | 0 |
| Inhibition parameter γ | | | | | |
| Layer | Feature | Letter | Orthographic | Associative | Response |
| Feature | 0 | -.15 | 0 | 0 | 0 |
| Letter | 0 | 0 | -.04 | 0 | 0 |
| Orthographic | 0 | 0 | -.21 | 0 | 0 |
| Associative | 0 | 0 | 0 | -.04 | 0 |
| Response | 0 | 0 | 0 | 0 | β |
| Note: β parameter of the LCA was estimated from data | | | | | |

| Table 2. Decay parameters | |
| --- | --- |
| Layer | AROM |
| Feature | .00 |
| Letter | .07 |
| Orthographic | .07 |
| Associative | .07 |
| Response | κ |
| Note: κ parameter of the LCA was estimated from data | |

| Table 3. Spearman correlations between model parameters | | | | | | | | | |
| --- | --- | --- | --- | --- | --- | --- | --- | --- | --- |
|  | β | ξ | $\rho_{New}$ | $\rho_{Old}$ | NDT | $\theta_{New-High}$ | $\theta_{New-Low}$ | $\theta_{Old-High}$ | $\theta_{Old-Low}$ |
| κ | -.02 | -.33 | .01 | .16 | -.11 | .16 | .05 | -.08 | .23 |
| β |  | -.10 | .15 | -.19 | -.15 | -.13 | -.45* | .22 | -.01 |
| ξ |  |  | -.50** | .33 | .03 | 0 | .11 | -.22 | -.10 |
| $\rho_{New}$ |  |  |  | -.21 | .05 | -.18 | -.21 | .10 | -.12 |
| $\rho_{Old}$ |  |  |  |  | .16 | 0 | .16 | -.09 | .04 |
| NDT |  |  |  |  |  | -.09 | -.04 | -.27 | -.20 |
| $\theta_{New-High}$ |  |  |  |  |  |  | .22 | -.01 | .10 |
| $\theta_{New-Low}$ |  |  |  |  |  |  |  | .03 | .11 |
| $\theta_{Old-High}$ |  |  |  |  |  |  |  |  | .50** |
| Note: * *p*<.05, ** *p* <.01 | | | | | | | | | |


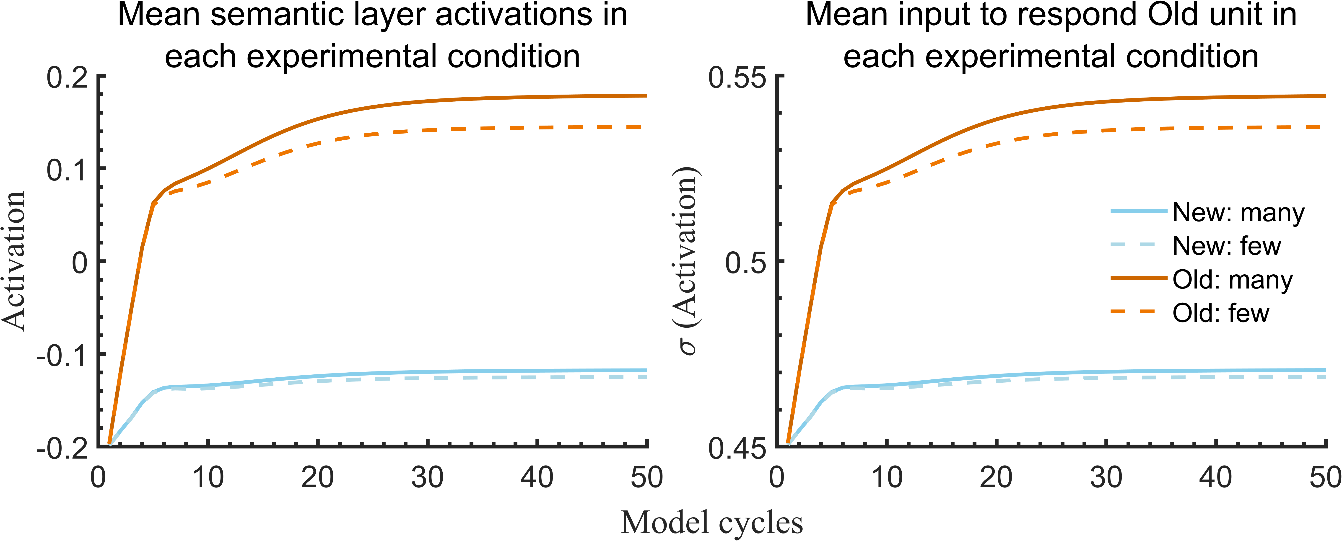


**Figure 1**. The **left** **plot** displays the effects of the resting levels and spreading activation in the four experimental conditions. The average activations of the semantic layer units are shown. The average was calculated over participants and items. The distance between blue and orange lines reflects the effect of higher resting levels for Old words. The differences between solid and dashed lines reflect the effect of spreading activation. The **right plot** shows the input to the Old response unit in the decision layer. That is, the values from the left are plotted after the sigmoid transformation has been applied. The patterns of activation from the semantic layer are preserved, but scaled so that the input for the LCA is positive. Both plots show that semantic many associates lead to a higher semantic activation.

# N400 linear mixed effects model regression tables

| Table 4. Random effects | | | |
| --- | --- | --- | --- |
| Groups | Name | Variance | SD |
| ID: Stimulus | Intercept | .4024 | .6343 |
| ID | Intercept | .1125 | .3354 |
|  | Residual | .2084 | .4565 |
| Number of obs.: 10911, groups: ID: Stimulus, 3637; ID, 29 | | | |

| Table 5. Fixed effects regression coefficients | | | |
| --- | --- | --- | --- |
| **Fixed Effect** | **β** | $\boldsymbol{SE}_{\boldsymbol{\beta}}$ | **t** |
| Intercept | .012 | .065 | .177 |
| D1 (Frontal vs. mean of Central and Posterior) | -.068 | .007 | -9.682 |
| D2 (Central vs. mean of Posterior and Frontal) | -.108 | .007 | -15.376 |
| Orthographic activation (Ortho) | -.010 | .013 | -.757 |
| AMSS | .093 | .014 | 6.842 |
| Criterion | .007 | .017 | .407 |
| Ortho: AMSS | -.010 | .014 | -.701 |
| Ortho: Criterion | .017 | .013 | 1.261 |
| AMSS: Criterion | .013 | .016 | .832 |
| D1: Ortho | -.009 | .007 | -1.258 |
| D2: Ortho | .003 | .007 | .469 |
| D1: AMSS | .023 | .007 | 3.331 |
| D2: AMSS | .005 | .007 | .706 |
| D1: Criterion | .046 | .007 | 6.153 |
| D2: Criterion | -.005 | .007 | -.703 |
| Ortho: AMSS: Criterion | -.010 | .013 | -.791 |
| D1: Ortho: AMSS | .002 | .007 | .239 |
| D2: Ortho: AMSS | .001 | .007 | .169 |
| D1: Ortho: Criterion | .006 | .007 | .844 |
| D2: Ortho: Criterion | .002 | .007 | .331 |
| D1: AMSS: Criterion | .006 | .007 | .873 |
| D2: AMSS: Criterion | -.002 | .007 | -.339 |
| D1: Ortho: AMSS: Criterion | .010 | .007 | 1.364 |
| D2: Ortho: AMSS: Criterion | -.003 | .007 | -.460 |
| Note: rlmer formula:  Amplitude ~ EEG Region*Ortho*AMSS* Criterion + (1\|ID) + (1\|ID:Stimulus);  D1, D2: dummy variables 1 and 2 | | | |

## N400 linear mixed effects model without mean orthographic activation

| Table 6. ANOVA style main and interaction effects | | | |
| --- | --- | --- | --- |
| **Effect** | **df** | **F-value** | ***p* - value** |
| Intercept | (1, 28.769) | .013 | .9092 |
| EEG Region | (2, 7266.000) | **193.662** | <.0001 |
| AMSS | (1, 3632.618) | **28.561** | <.0001 |
| Criterion | (1, 3410.987) | .231 | .6306 |
| EEG Region: AMSS | (2, 7266.000) | **5.325** | .0049 |
| EEG Region: Criterion | (2, 7266.000) | **10.475** | <.0001 |
| AMSS: Criterion | (1, 3493.075) | 1.080 | .2987 |
| EEG Region: AMSS: Criterion | (2, 7266.000) | .950 | .3866 |
| Notes:  rlmer formula: Amplitude ~ EEG Region * AMSS * Criterion + (1\|ID) + (1\|ID:Stimulus);  *p*-values were obtained using the Kenward-Roger approximation. Significant F-values are marked in bold | | | |

| Table 7. Random effects of the regression model | | | |
| --- | --- | --- | --- |
| Groups | Name | Variance | SD |
| ID: Stimulus | Intercept | .4022 | .6342 |
| ID | Intercept | .1124 | .3353 |
|  | Residual | .2086 | .4568 |
| Number of obs: 10911, groups: ID: Stimulus, 3637; ID, 29 | | | |

| Table 8. Fixed effects regression coefficients | | | |
| --- | --- | --- | --- |
| **Fixed Effect** | **β** | $\boldsymbol{SE}_{\boldsymbol{\beta}}$ | **t** |
| Intercept | .011 | .065 | .169 |
| D1 (Frontal vs. mean of Central and Posterior) | -.068 | .007 | -9.654 |
| D2 (Central vs. mean of Posterior and Frontal) | -.108 | .007 | -15.408 |
| AMSS | .092 | .014 | 6.812 |
| Criterion | .006 | .017 | .352 |
| D1: AMSS | .023 | .007 | 3.206 |
| D2: AMSS | .005 | .007 | .761 |
| D1: Criterion | .046 | .007 | 6.223 |
| D2: Criterion | -.005 | .007 | -.728 |
| AMSS: Criterion | .015 | .016 | .902 |
| D1: AMSS: Criterion | .007 | .007 | .980 |
| D2: AMSS: Criterion | -.002 | .007 | -.334 |
| Note: rlmer formula: Amplitude ~ EEG Region*AMSS* Criterion + (1\|ID) + (1\|ID:Stimulus)  D1, D2: dummy variables 1 and 2 | | | |

# N400 linear mixed effects model summary for measures of orthographic and associative activation from the last seven processing cycles

| Table 9. Main and interaction effects for the N400 linear mixed effects model | | | |
| --- | --- | --- | --- |
| **Effect** | ***df*** | **F-value** | ***p* - value** |
| Intercept | (1, 28.784) | .0186 | .8925 |
| EEG Region | (2, 7258.000) | **187.807** | <.0001 |
| Orthographic activation (Ortho) | (1, 3602.660) | .952 | .3292 |
| AMSS | (1, 3628.430) | **32.682** | <.0001 |
| Criterion | (1, 3427.030) | .097 | .7560 |
| EEG Region: Ortho | (2, 7258.000) | 2.372 | .0933 |
| EEG Region: AMSS | (2, 7258.000) | **4.726** | .0089 |
| Ortho: AMSS | (1, 3602.340) | 1.333 | .2483 |
| EEG Region: Criterion | (2, 7258.000) | **10.469** | <.0001 |
| Ortho: Criterion | (1, 3602.360) | .999 | .3174 |
| AMSS: Criterion | (1, 3481.760) | 1.178 | .2778 |
| EEG Region: Ortho: AMSS | (2, 7258.000) | .669 | .5125 |
| EEG Region: Ortho: Criterion | (2, 7258.000) | .954 | .3851 |
| EEG Region: AMSS: Criterion | (2, 7258.000) | 1.008 | .3651 |
| Ortho: AMSS: Criterion | (1, 3602.100) | .075 | .7835 |
| EEG Region: Ortho: AMSS: Criterion | (2, 7258.000) | .658 | .5181 |
| Notes:  rlmer formula: Amplitude ~ EEG Region * Ortho * AMSS * Criterion + (1\|ID) + (1\|ID:Stimulus);  *p*-values were obtained using the Kenward-Roger approximation. Significant F-values are marked in bold. | | | |

# EEG preprocessing

We first reduced the sampling rate to 250 Hz and then removed line noise at 50Hz using the CleanLine plug-in for EEGLAB. Next, the data were high pass filtered using a 1Hz threshold. We then decomposed the data using independent component analysis as implemented in the EEGLAB infomax picard routine. The extracted components were then applied to the unfiltered data and labeled using the ICLabel EEGLAB plug-in. After labelling, components identified as eye, muscle or line noise were inspected and removed from the data. We also inspected the components classified as other to identify further noise sources and remove them. The cleaned data were then filtered using a band-pass filter keeping frequencies 0.1 and 30Hz. Next steps were performed in ERPLAB. First, epochs were defined, extracted, and visually inspected for any residual artifacts. If needed channels were interpolated using EEGLAB routines (in total three channels in two subjects).

# ERP plots and mean N400 amplitudes


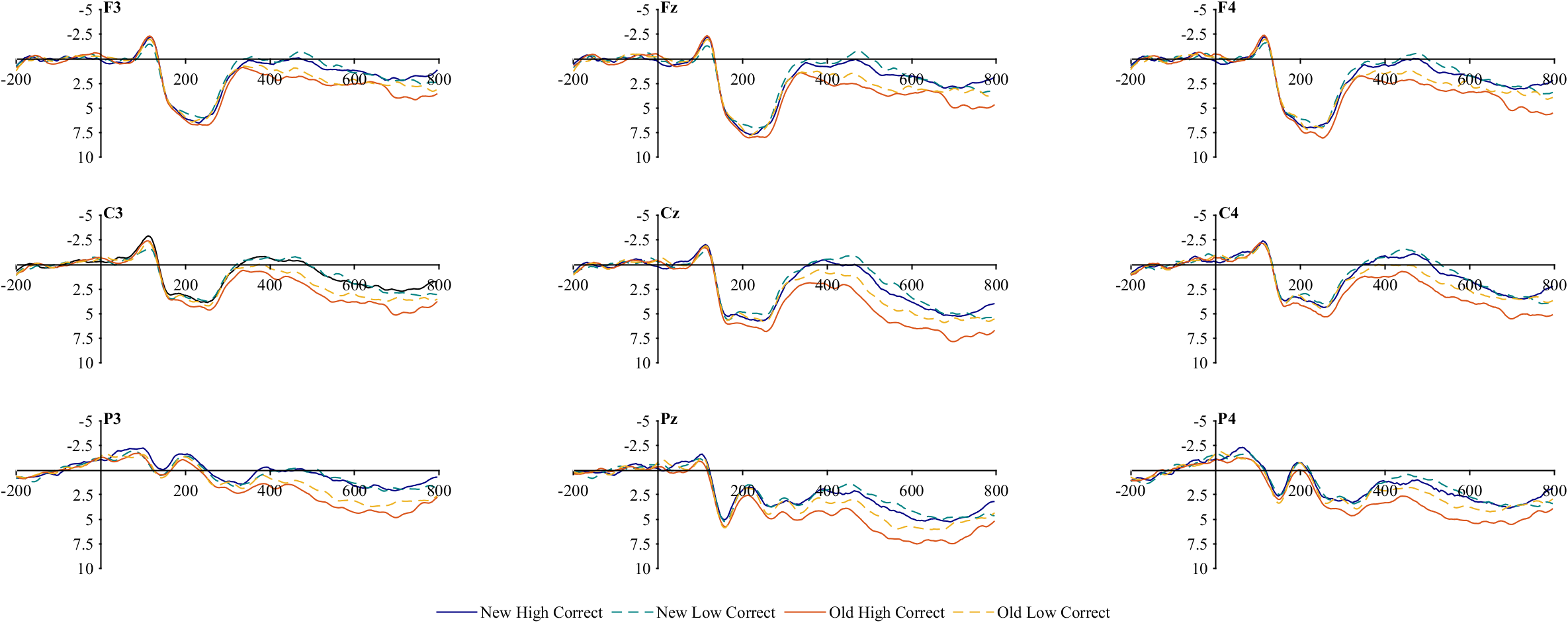


Figure 2. ERPs for the analyzed electrodes.


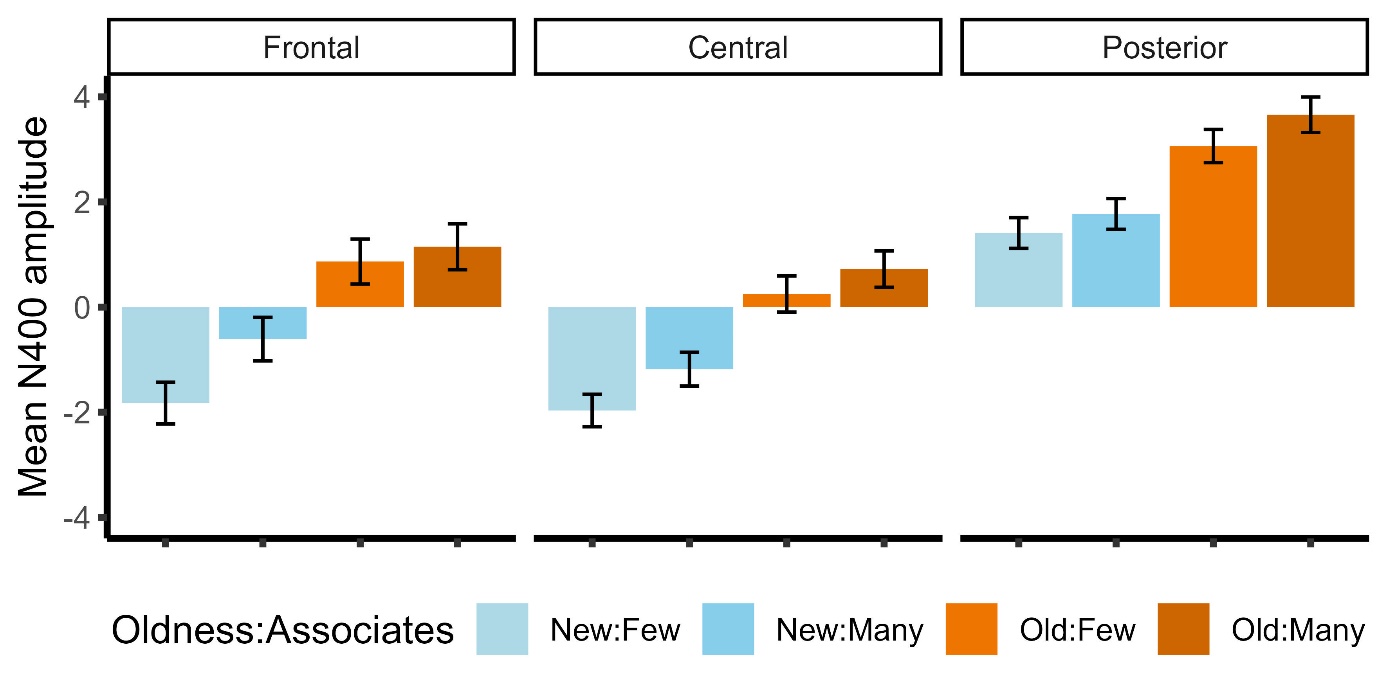


Figure 3. Mean N400 amplitudes and standard errors of the mean for each experimental condition (Oldness: Number of associates) for each EEG region.

# AROM+ with a single criterion

The only difference compared to the model in the main text is that there is only one criterion.


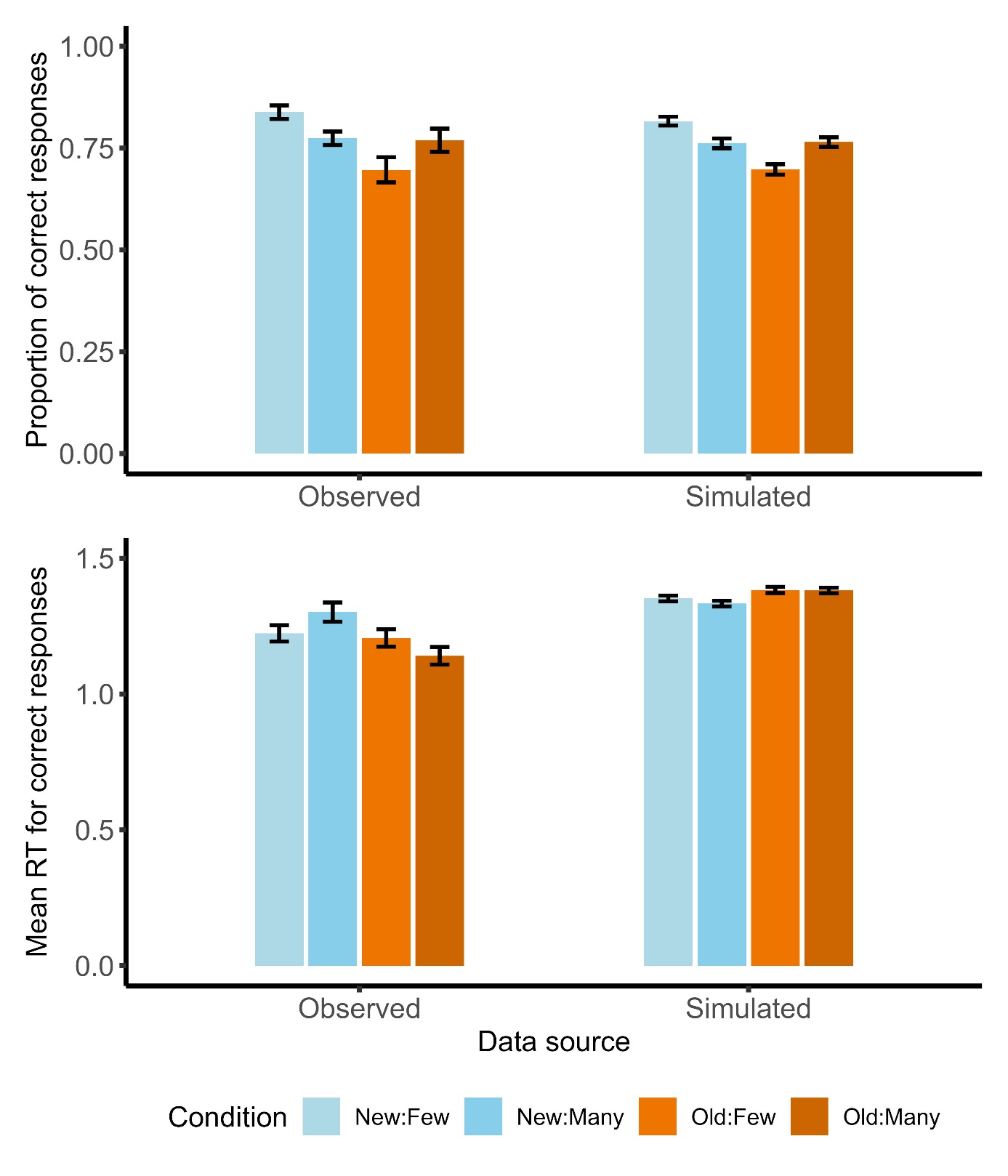


Figure 4. Performance (means and standard errors) in the four experimental conditions (Oldness: Number of associates) from behavioral and simulated data. **a**: average proportion of correct responses (hits and correct rejections). **b**: average response times in seconds for correct responses (hits and correct rejections). While the single response criterion model can capture accuracy, it fails to reproduce response times.
